# Supplementary material for: An amphioxus neurula stage cell atlas supports a complex scenario for the emergence of vertebrate head mesoderm
Source: Nat Commun. 2024 May 29;15:4550. doi: 10.1038/s41467-024-48774-4 (PMC11136973; doi:10.1038/s41467-024-48774-4)
Supplement: Supplementary file 1 — Supplementary Information [file 41467_2024_48774_MOESM1_ESM.pdf]

An amphioxus neurula stage cell atlas supports a complex scenario for the emergence of vertebrate head mesoderm

Xavier Grau-Bové[1,*], Lucie Subirana[2,*], Lydvina Meister[2], Anaël Soubigou[2], Ana Neto[3], Anamaria Elek[1,4], Silvia Naranjo[3], Oscar Fornas[5,6], Jose Luis Gomez-Skarmeta[3], Juan J. Tena[3], Manuel Irimia[1,4,7], Stéphanie Bertrand[2,8,#], Arnau Sebé-Pedrós[1,4,7,#], Hector Escriva[2,#]

1. Centre for Genomic Regulation (CRG), Barcelona Institute of Science and Technology (BIST), Barcelona, Spain.
2. Sorbonne Université, CNRS, Biologie Intégrative des Organismes Marins, BIOM, F-66650, Banyuls-sur-Mer, France.
3. Centro Andaluz de Biología del Desarrollo (CABD), CSIC-Universidad Pablo de Olavide-Junta de Andalucía, Sevilla, Spain
4. Universitat Pompeu Fabra (UPF), Barcelona, Spain.
5. Flow Cytometry Unit, Centre for Genomic Regulation (CRG), The Barcelona Institute for Science and Technology (BIST), Barcelona, Spain.
6. Departament de Ciències Experimentals i de la Salut, Universitat Pompeu Fabra (UPF), Barcelona, Spain.
7. ICREA, Barcelona, Spain.
8. Institut universitaire de France (IUF), Paris, France

*Contributed equally

#Corresponding authors: Hector Escriva (hescriva@obs-banyuls.fr), Arnau Sebé-Pedrós (arnau.sebe@crg.eu), Stéphanie Bertrand (stephanie.bertrand@obs-banyuls.fr)

This file includes:

- **Supplementary Figure 1. scRNA-seq and ATAC-seq summary statistics (related to Fig. 1 and 3)**
- **Supplementary Figure 2. *In situ* hybridization of genes showing an enriched expression in some metacells and/or for which expression was not previously described**
- **Supplementary Figure 3**
- **Supplementary Figure 4. Transcription factor expression and motif activity (related to Fig. 1 and Fig. 3)**
- **Supplementary Figure 5. Gene expression distribution on 2D projected cells for neural gene markers**
- **Supplementary Figure 6. Gene expression distribution on 2D projected cells for endodermal gene markers (related to Fig. 4)**
- **Supplementary Figure 7. Gene expression distribution on 2D projected cells for somitic gene markers (related to Fig. 5)**
- **Supplementary Figure 8. Analysis of the activity of putative regulatory regions of amphioxus genes in zebrafish**
- **Supplementary Note**
- **Supplementary References**

Supplementary Figure 1

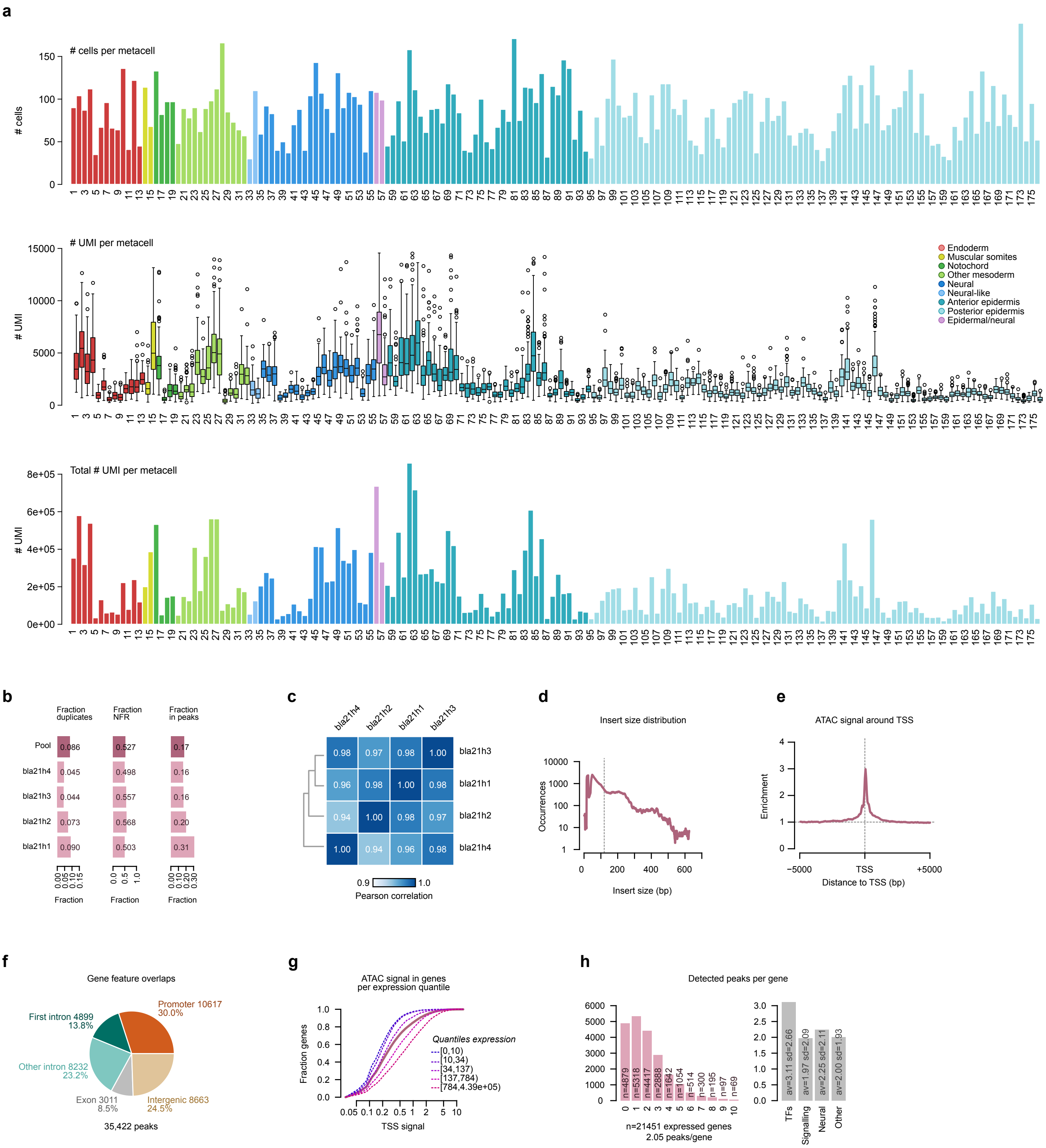

Supplementary Figure 1. **scRNA-seq and ATAC-seq summary statistics (related to Fig. 1 and 3). a,** Number of cells per metacell cluster. distribution of UMIs/cell in each metacell, and total number of UMIs per metacell. **b,** Fraction of reads in each ATAC-seq sample (and the pooled dataset) that are duplicated, nucleosome-free (NFR), or mapping in peaks. **c,** Inter-sample similarity for the ATAC-seq replicates, measured using the Pearson correlation coefficient of binned raw counts in the nucleosome-free fraction (bin size = 10 kbp). **d,** Insert size distribution of the pool of ATAC-seq replicates. The dotted line indicates the threshold to define the nucleosome-free fraction (120 bp). **e,** Enrichment of ATAC-seq signal around transcription start sites (TSS), calculated using binned normalised coverage (bin size = 50 bp). **f,** Fraction of ATAC-seq peaks overlapping various features in the genome. **g,** Cumulative distribution of the normalised ATAC-seq signal at the TSS of genes, sorted in five equally-sized bins according to their expression levels (low to high, measured in UMI counts). Highly expressed genes in our scRNA-seq data exhibit stronger bulk ATAC-seq signals. **h,** Distribution of number of ATAC-seq peaks detected per gene, in global (left) and for specific subsets of gene families (TFs, signalling-related genes, and neural-related genes; right).

Supplementary Figure 2

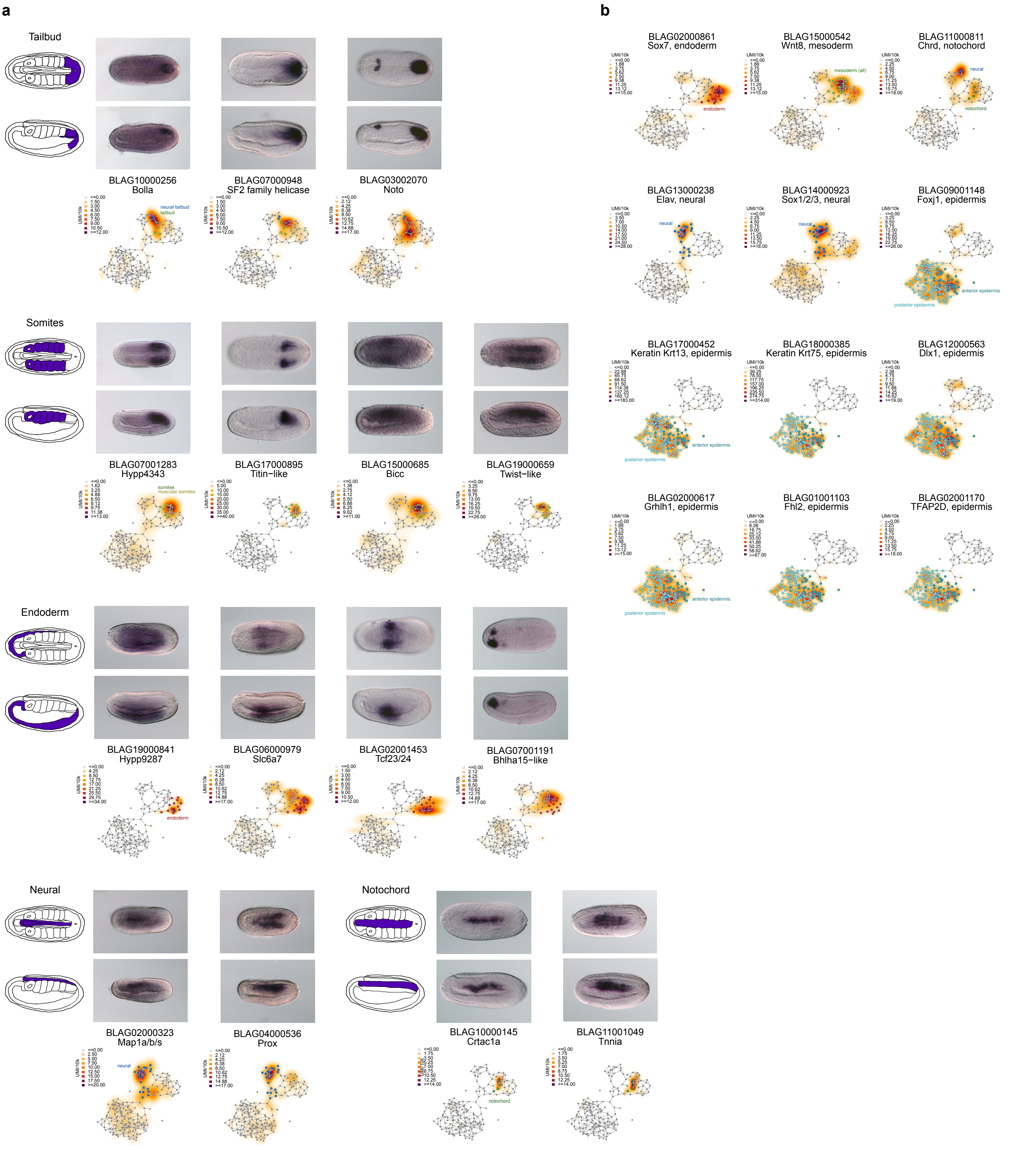

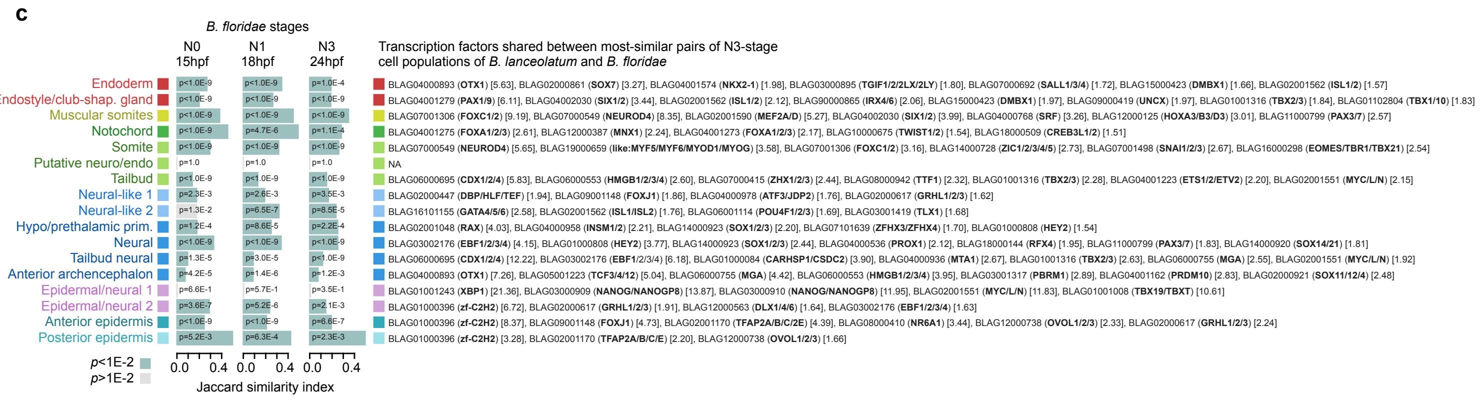

Supplementary Figure 2. *In situ* hybridization of genes showing an enriched expression in some metacells and/or for which expression was not previously described. a, *in situ* hybridization experiments in N3 stage embryos. Dorsal views with anterior to the left (top) and side views with anterior to the left and dorsal to the top are shown for each gene. Schemes of embryo showing in blue the region in which each series of genes is expressed is presented on the left. Below each *in situ* hybridization picture, transcriptomic expression of the marker is shown as density maps representing UMI counts (per 10,000 UMIs) in each cell, using the same two-dimensional metacell arrangement as in Fig. 1. **b,** Expression profile of additional lineage markers for endoderm, mesoderm, notochord, neural and epidermis, taken from[1]. **c,** Validation of the cell clusters described in our *B. lanceolatum* dataset by identification of matching cell clusters in three neurula stages (N0, N1, and N3) in another amphioxus species, *B. floridae*. For each *B. lanceolatum* cell type, we report the TF usage overlap (as Jaccard index) with the top-ranking metacell in each *B. floridae* stage. The sets of cluster-specific TFs in each species were selected based on fold-change overexpression (FC $\geq$ 1.5), and *p*-values on the Jaccard index values calculated using the Measure Concentration Algorithm.

Supplementary Figure 3

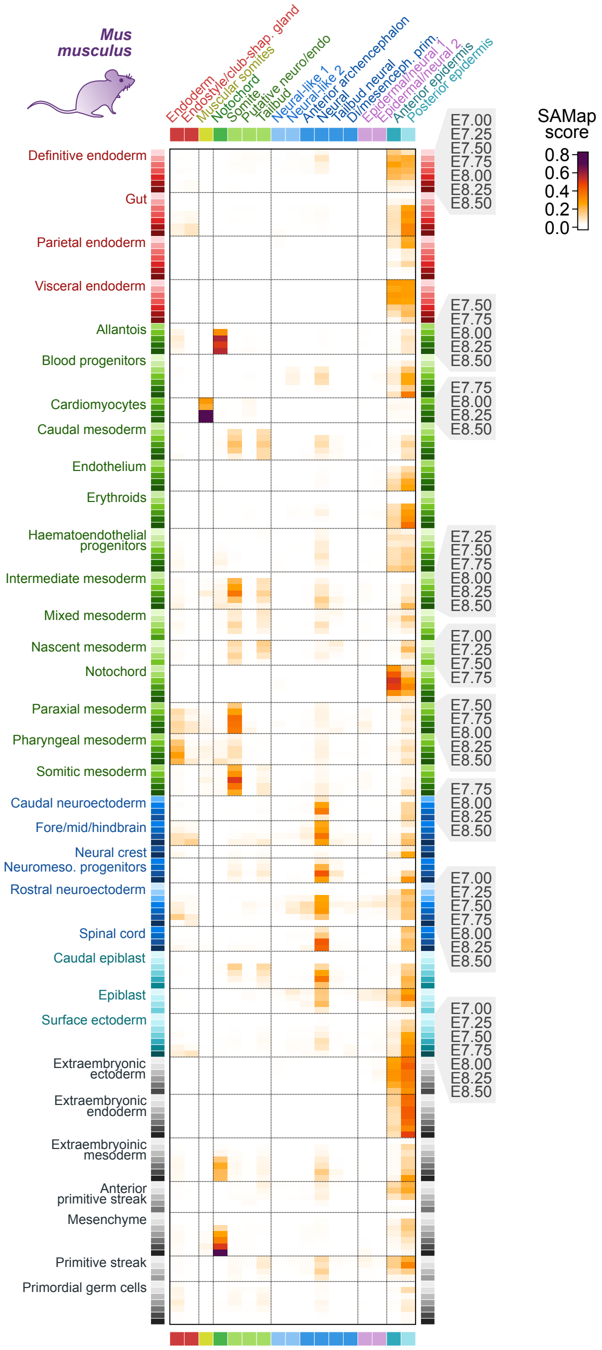

Supplementary Figure 3. Cross-species comparison of *B. lanceolatum* (columns) with a *Mus musculus* developmental timecourse (rows, E7.0 to E8.5). Cell type similarity was measured using SAMap scores based on all available pairwise markers (see *Methods*). Cell types are colour-coded by cell type or developmental layer (endoderm, mesoderm/muscle, neuroectoderm, ectoderm, and other), and, in the case of the multi-stage chordate datasets, by developmental time-point (colour intensity, legend to the side of each plot)

Supplementary Figure 4

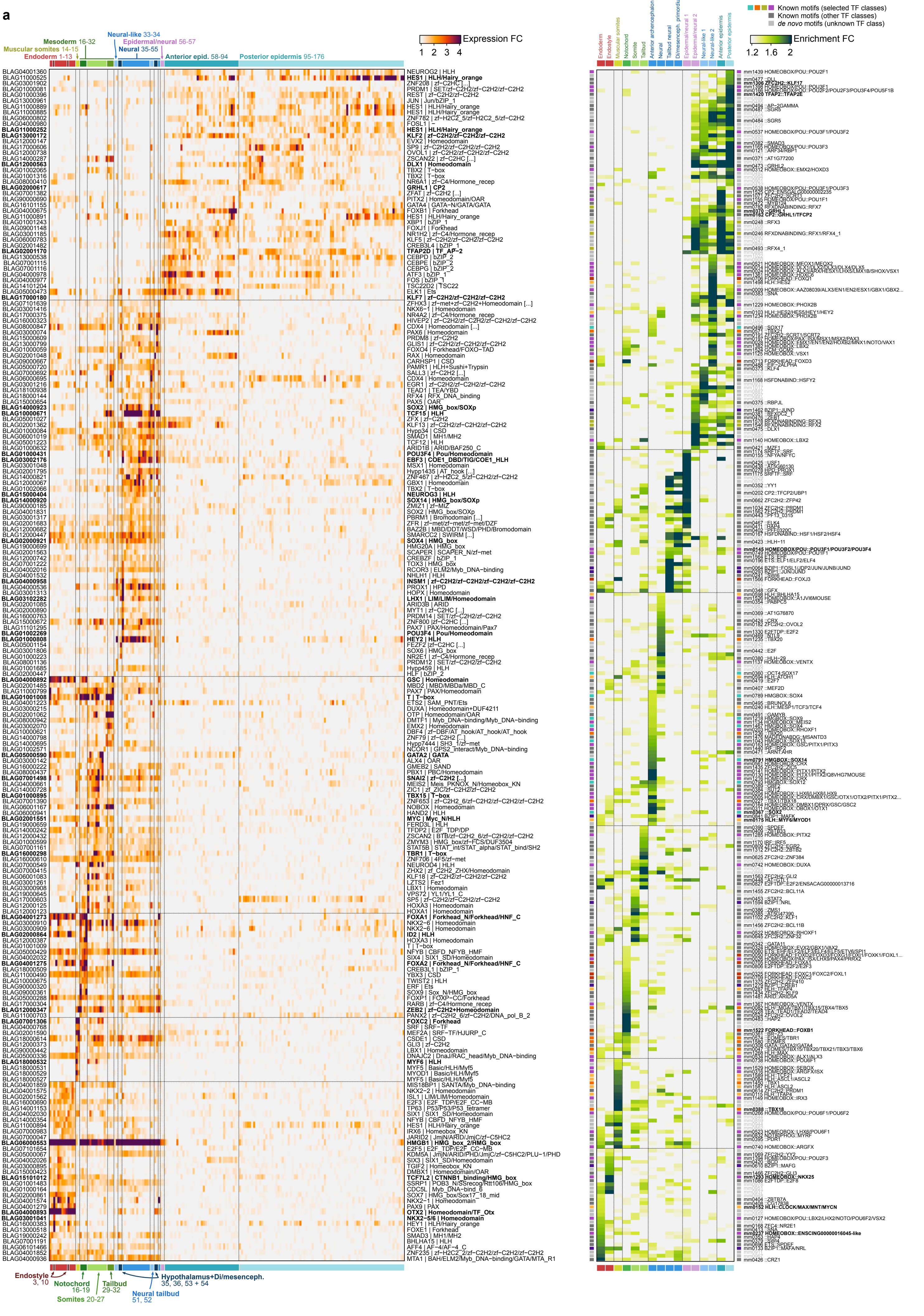

Supplementary Figure 4. Transcription factor expression and motif activity (related to Fig. 1 and Fig. 3). a, Normalized fold change expression of top variable TFs (rows) per metacell (columns, grouped by cell type). For each metacell, we selected TFs with a minimum fold change ≥ 2 and a total of 10 UMIs across all cells. Gene names in bold indicate that the gene is mentioned in the manuscript. **b,** Enrichment fold change of top variable TF binding motifs (rows) per cell type (columns). For each cell type, we selected up to 60 motifs with a minimum fold change ≥ 1.2 and enrichment BH-adjusted *p*-value < 0.05. Motifs are color-coded based on their sequence similarity to motifs of known TF structural classes (see *Methods*): light gray indicates *de novo* motifs without similar motifs in known databases, whereas dark gray and other colors indicate motifs that can be mapped to one or more previously described TF binding motifs. Motifs in bold are mentioned in the manuscript.

Supplementary Figure 5

a

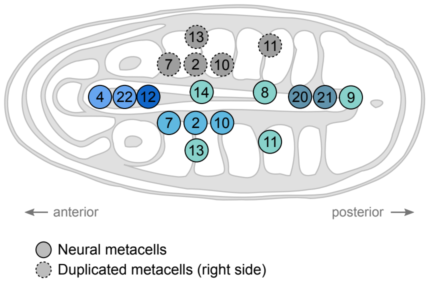


```
    annotation
2   anterior spinal chord segmentally arranged neurons
4   rostral anterior archencephalon (cerebral vesicle)
7   anterior spinal chord segmentally arranged neurons
8   posterior floorplate
9   tailbud floorplate
10  median pair of neurons
11  posterior neural plate border
12  di-mesencephalic primordium (cerebral vesicle)
13  anterior neural plate border
14  floorplate
20  tailbud neural
21  tailbud neural
22  anterior archencephalon (cerebral vesicle)

Unclassified: 1,3,5,6,15-19,23-25
```

b

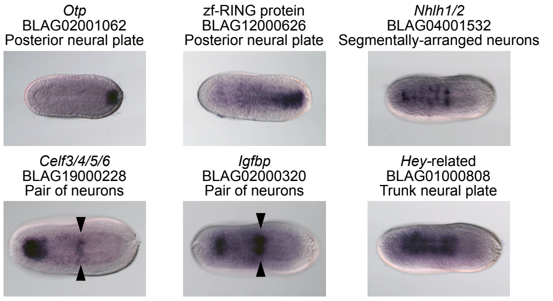

c

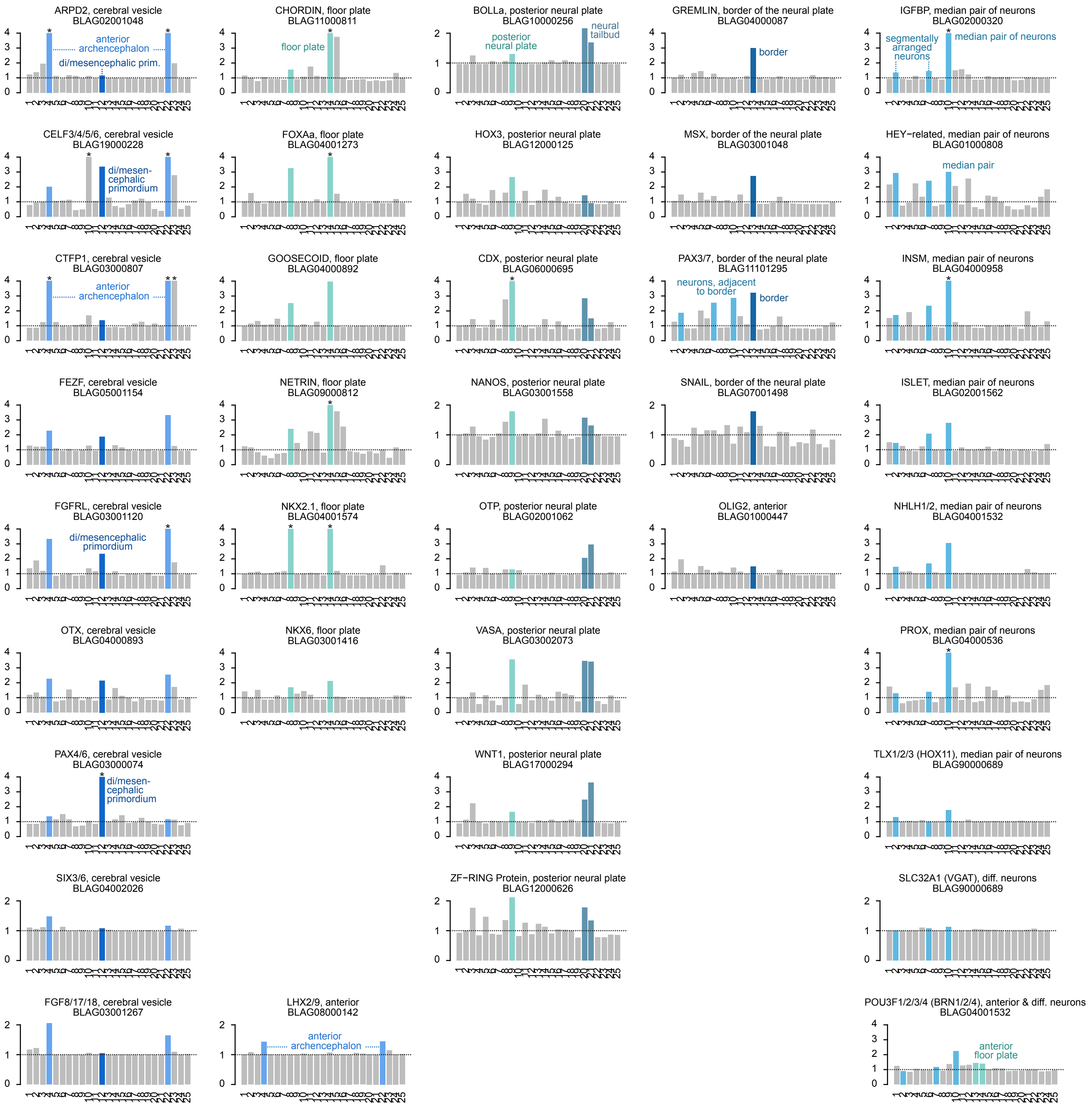

Supplementary Figure 5. Gene expression distribution on 2D projected cells for neural gene markers. a, Schematics of inferred neural metacell locations over an amphioxus neurula-stage embryo, dorsal with anterior to the left. Metacell annotations (right) are based on the expression of specific markers (panels b and c). **b,** ISH of selected neural markers. **c,** Normalized fold change expression of selected markers in the neural reclustering. Markers are arranged vertically according to similar expression profiles (e.g. the first column includes all markers linked to the presumptive cerebral vesicle), and metacells are highlighted accordingly. Markers were selected from the literature and from ISH analysis of newly discovered genes overexpressed in specific metacells in our dataset.

Supplementary Figure 6

a

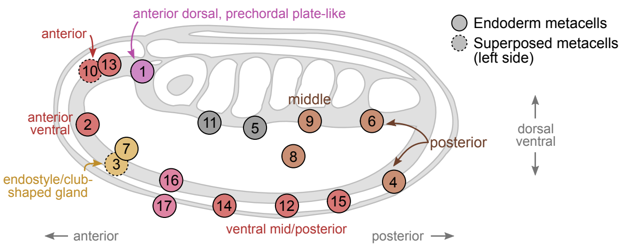

	annotation
1	prechordal plate-like territory; anterior dorsal endoderm
2	anterior ventral endoderm
3	left club-shaped gland/endostyle presumptive region
4	ventral posterior endoderm
5	dorsal mid-endoderm region
6	posterior endoderm
7	right club-shaped gland/endostyle presumptive region
8	mid/posterior endoderm
9	mid/posterior endoderm
10	Hatschek's diverticulum presumptive region; anterior endoderm
11	dorsal mid-endoderm region
12	ventral mid/posterior endoderm
13	Hatschek's diverticulum presumptive region; anterior endoderm
14	region posterior to the first pharyngeal slit presumptive territory
15	mid/posterior endoderm
16	first pharyngeal slit presumptive region
17	first pharyngeal slit presumptive region

b

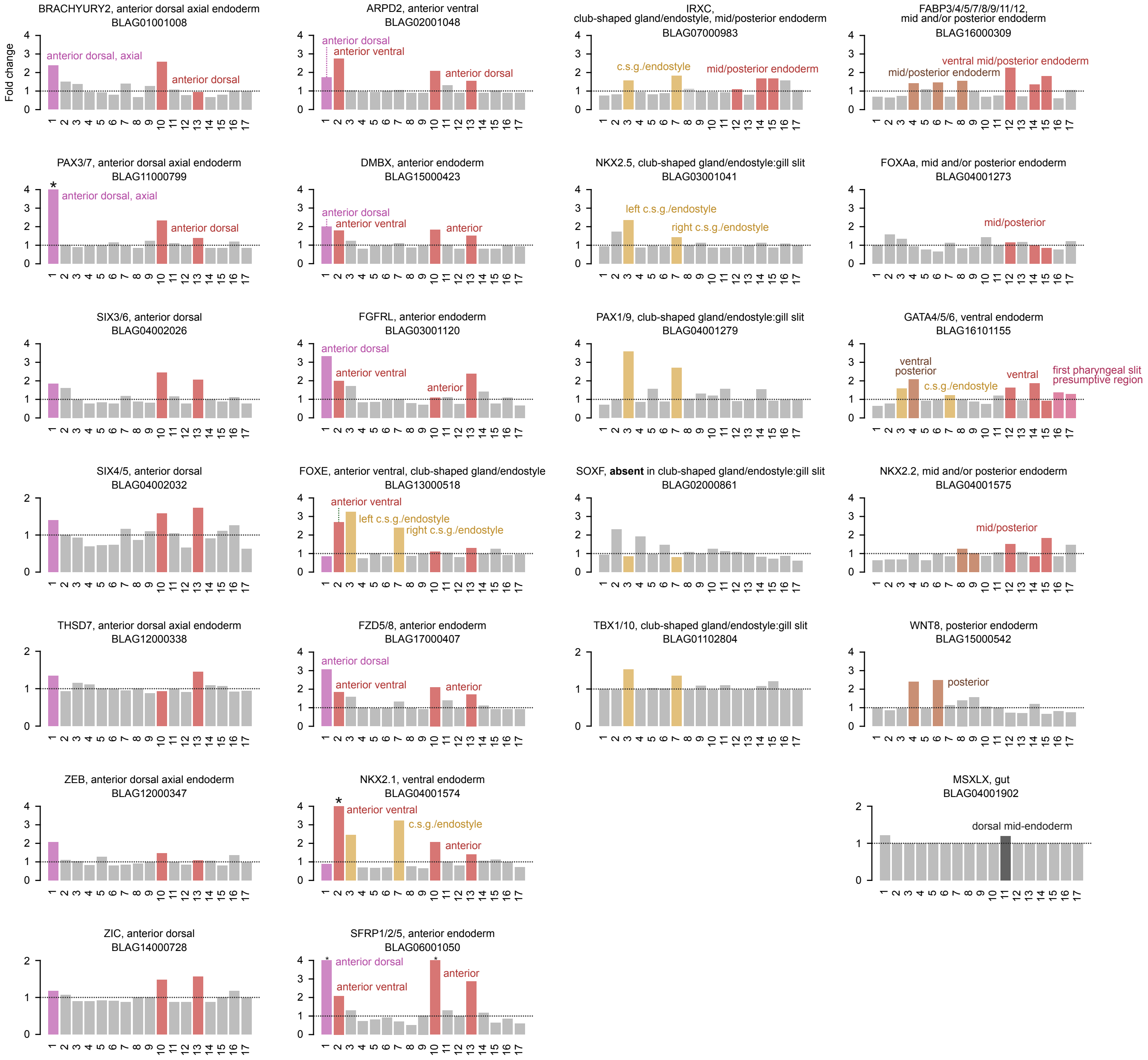

Supplementary Figure 6. Gene expression distribution on 2D projected cells for endodermal gene markers (related to Fig. 4). a, Schematics of inferred endodermal metacell locations over an amphioxus neurula-stage embryo, side view with anterior to the left and dorsal to the top. Metacell annotations (right) are based on the expression of specific markers (panel b). **b,** Normalized fold change expression of selected markers in the endodermal reclustering. Markers are arranged vertically according to similar expression profiles, and specific metacells are highlighted accordingly.

Supplementary Figure 7

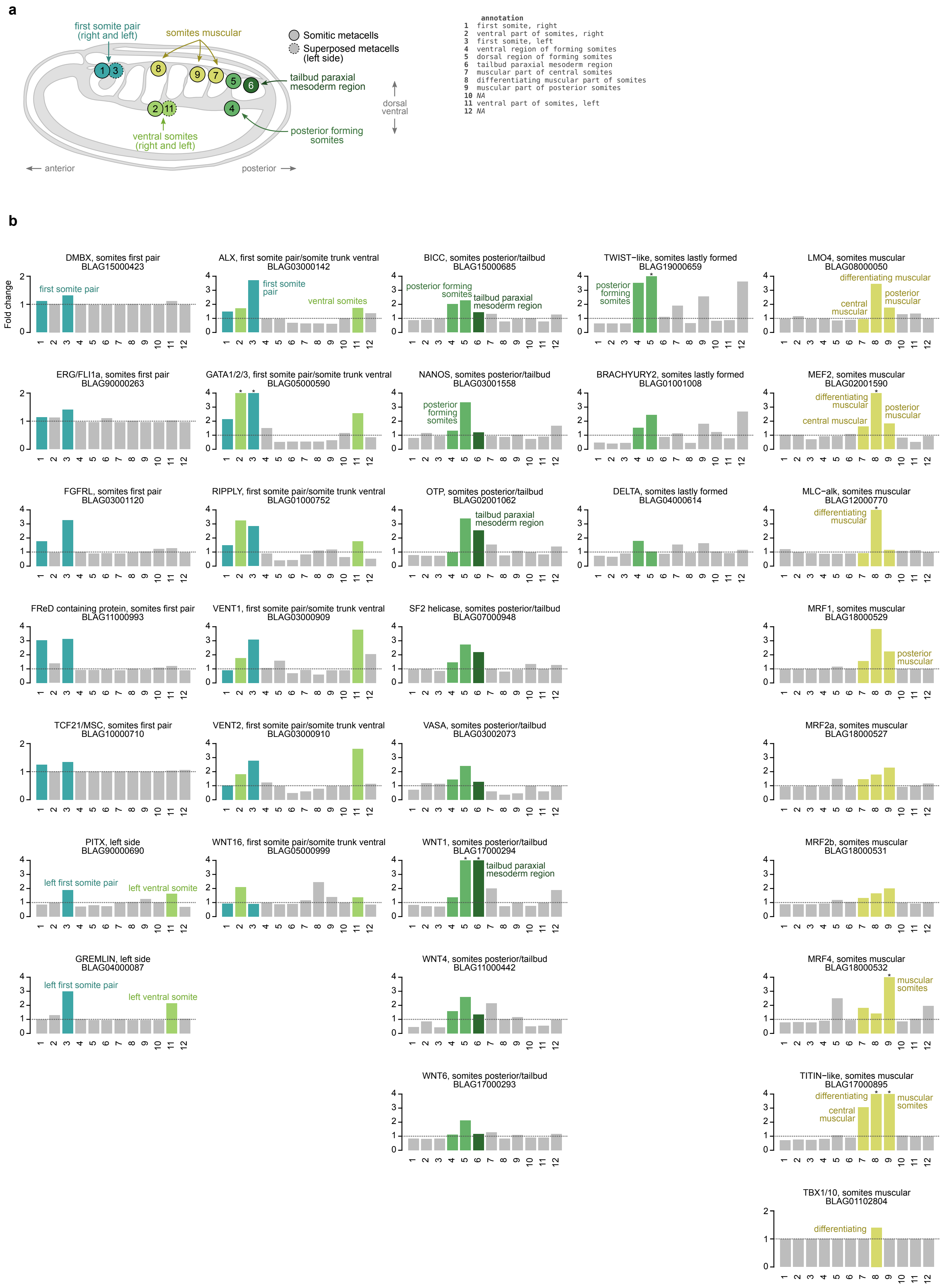

Supplementary Figure 7. Gene expression distribution on 2D projected cells for somitic gene markers (related to Fig. 5). a, Schematics of inferred somite metacell locations over an amphioxus neurula-stage embryo, side view with anterior side to the left and dorsal to the top. Metacell annotations (right) are based on the expression of specific markers (panel b). **b,** Normalized fold change expression of selected markers in the somitic reclustering. Markers are arranged vertically according to similar expression profiles, and specific metacells are highlighted accordingly.

Supplementary Figure 8

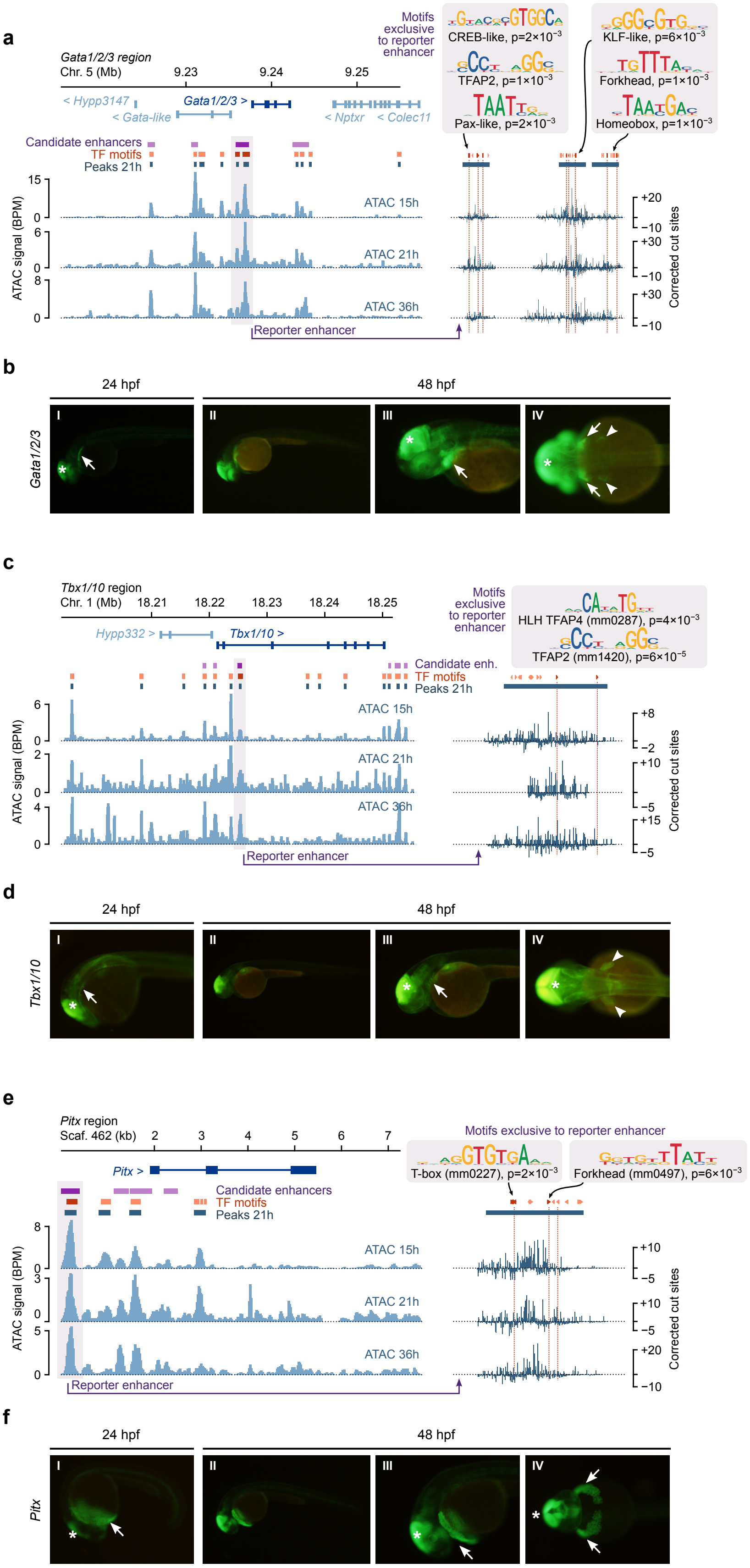

Supplementary Figure 8. Analysis of the activity of putative regulatory regions of amphioxus genes in zebrafish. a, Identification of putative enhancers of *Gata1/2/3* in amphioxus, based on the examination of bulk ATAC-seq experiments (at 15 hpf, 21 hpf, and 36 hpf, measured in bins per million mapped reads, or BPM). ATAC-seq peaks at 21 hpf are showed in dark grey. Candidate enhancer regions are shown in purple. Mapped TF motifs are shown in red. The right panel to the right shows a zoom-in of the enhancer region cloned in the reporter construct in panel b (grey-shaded region), highlighting some of its unique TF motifs (top, *p*-values reflect significance of enrichment of the motif in that genomic window; see *Methods* and Supplementary Data 2 for the complete list) and the TF binding signatures for each ATAC-seq library (expressed as *TOBIAS*-corrected ATAC cut sites, where negative values indicate regions that are putatively bound by a protein). **b**, GFP signal in F1 transgenic zebrafish embryos for the *Gata1/2/3* construct. Subpanels I to III show the lateral view (anterior to the left) of 24 hpf or 48 hpf embryos showing green fluorescence in the pharyngeal mesoderm (arrows). Subpanel IV shows a dorsal view of the same 48 hpf individual from panel III with green fluorescence in the fin buds (arrowheads). The fluorescence observed in the midbrain corresponds to the positive control included into the reporter constructs and is indicated by a white asterisk. **c,** Same as panel a, indicating putative enhancers of *Tbx1/10* in amphioxus (left) and the unique motifs and TF binding signatures of the reporter enhancer (right). **d,** Same as panel b, showing green fluorescence in the pharyngeal mesoderm from lateral viewpoints (arrows, subpanels I to III) and fin buds from a dorsal viewpoint (arrowheads, subpanel IV), at different developmental stages (24 and 48 hpf). **e,** Same as panels a and c, indicating putative enhancers of *Pitx* (left) and the unique motifs and TF binding signatures of the reporter enhancer (right). **f,** Same as panels b and d, showing green fluorescence in the hatching gland cells from lateral (arrows, subpanels I to III) and ventral (IV) viewpoints, at different developmental stages (24 and 48 hpf).

Supplementary Figure 9

a. *Gata1/2/3*

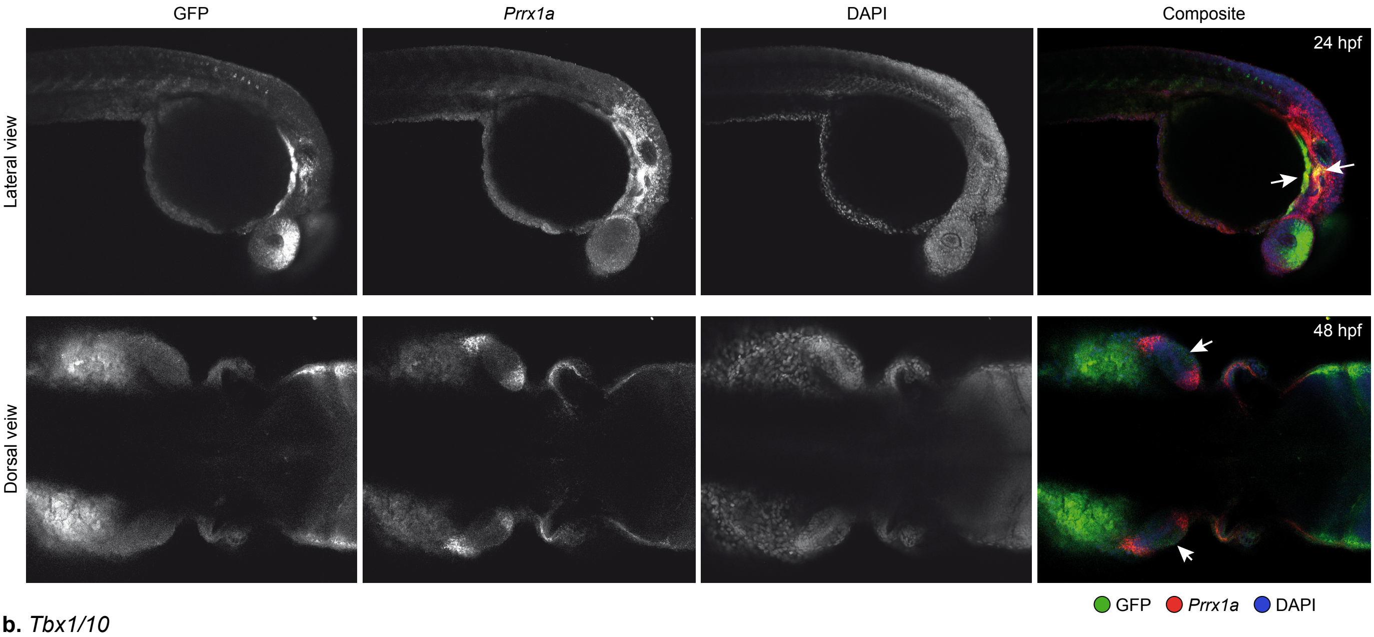

b. *Tbx1/10*

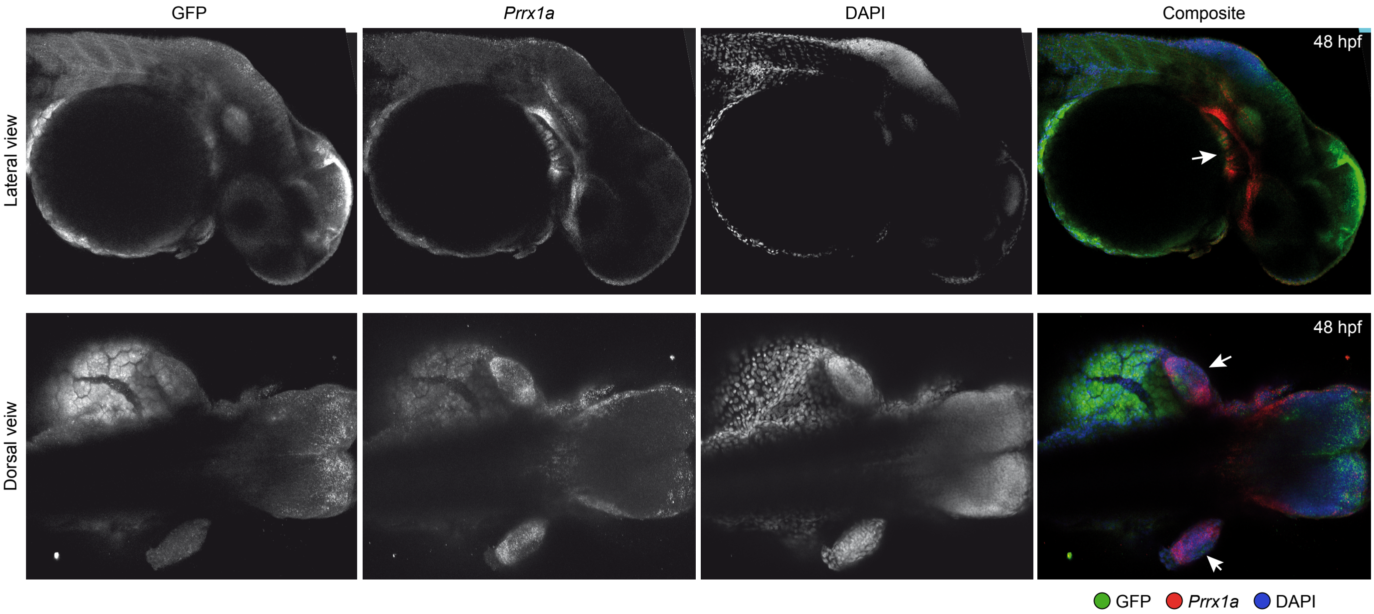

Supplementary Figure 9. Co-expression of *GFP* and *Prrx1a* in transgenic zebrafish embryos. HCR *in situ* hybridization in embryos at 24 hpf and/or 48 hpf of the transgenic lines with the reporter constructs for *Gata1/2/3* (**a**) and *Tbx1/10* (**b**). Lateral views with anterior to the right and dorsal to the top are shown, as well as dorsal views with anterior to the left. For the *Gata1/2/3* construction transgenics (**a**), coexpression of *Prrx1a* and *GFP* is shown at 24 hpf in part of the head mesenchyme (arrows) and at 48 hpf in the finbuds (arrows). For the *Tbx1/10* construction transgenics (**b**), coexpression was observed at 48 hpf in part of the head mesenchyme (arrows in side view) and in the finbuds (arrows in dorsal view).

Supplementary Note:

Description of the neural sub-clustering data

Neural plate cells could be clustered into 22 metacells (Supplementary Fig. 5). Among these, we recognized three metacells corresponding to the presumptive cerebral vesicle (4, 12, and 22). Metacells 4 and 22 coexpress *Arpd2*, *Fezf*, *Fgfrl*, *Fgf8/17/18*, and *Otx*[2-5] (Supplementary Fig. 5) together with *Celf3/4/5/6*[6] (Supplementary Fig. 5a, b) and *Ctfp1* (Fig. 1e, Supplementary Fig. 5) and correspond to the anterior archencephalon, a region previously named Hypothalamo-prethalamic primordium in[2] with metacell 4 overexpressing *Six3/6*[2] (Supplementary Fig. 5) and hence representing its rostral part. On the other hand, metacell 12 shows expression of *Otx* and *Pax4/6*, a combination typical of the Di-Mesencephalic primordium[2] (Supplementary Fig. 5). Metacells 20 and 21 co-express the posterior gene markers *Cdx*, *Nanos*, *Otp*, *Vasa* and *Wnt1*[2,7-9] (Supplementary Fig. 5), together with *Bolla* and *Zf-Ring Protein* described here (Supplementary Fig. 2a, Supplementary Figure 5), suggesting that these metacells represent the posterior-most neural plate. In addition to expressing posterior markers, metacell 9 also expresses *Netrin* that marks the floorplate[10] (Supplementary Fig. 5). According to the expression of the floor plate marker genes *Chordin*, *Foxaa*, *Goosecoid*, *Netrin*, *Nkx2.1* and *Nkx6*[2,10-16] (Supplementary Fig. 5), metacells 8 and 14 could be assigned to this structure, with metacell 8 additionally expressing the posterior genes *Cdx* and *Hox3*[2,7,17] (Supplementary Fig. 5). The expression of *Msx*, *Pax3/7* and *Snail*[2,18-20] in metacells 11 and 13 indicate they belong to the neural plate border with metacell 13 expressing the anterior marker *Gremlin*[21], and metacell 11 expressing the posterior gene *Hox3*[2,17] (Supplementary Fig. 5a). We could also recognize metacells 2, 7 and 10 as segmentally arranged neurons co-expressing *Islet*[22] (Supplementary Fig. 5) and *Nhlh1/2* (Supplementary Fig. 5a,b), with metacell 10 corresponding to a specific pair of neurons characterized by *Celf3/4/5/6* and *Igfbp* expression (Supplementary Fig. 5a,b). All the other metacells show few specific markers and could represent differentiating cells. These cells express different combinations of the known neural genes *Elav*[23] and *Neurogenin*[24], together with *Hey-related* (Supplementary Fig. 5b), *Prox* (Supplementary Fig. 2, 5) and *Tcf15-like* genes (Fig. 1e, Supplementary Fig. 5).

Supplementary References

1 Ma, P. *et al.* Joint profiling of gene expression and chromatin accessibility during amphioxus development at single-cell resolution. *Cell Reports* **39**, 110979, doi:https://doi.org/10.1016/j.celrep.2022.110979 (2022).

2 Albuixech-Crespo, B. *et al.* Molecular regionalization of the developing amphioxus neural tube challenges major partitions of the vertebrate brain. *PLoS Biol* **15**, e2001573, doi:10.1371/journal.pbio.2001573 (2017).

3 Bertrand, S. *et al.* Amphioxus FGF signaling predicts the acquisition of vertebrate morphological traits. *Proc Natl Acad Sci U S A* **108**, 9160-9165, doi:10.1073/pnas.1014235108 (2011).

4 Bertrand, S., Somorjai, I., Garcia-Fernandez, J., Lamonerie, T. & Escriva, H. FGFRL1 is a neglected putative actor of the FGF signalling pathway present in all major metazoan phyla. *BMC Evolutionary Biology* **9**, 226, doi:10.1186/1471-2148-9-226 (2009).

5 Williams, N. A. & Holland, P. W. Old head on young shoulders. *Nature* **383**, 490-490 (1996).

6 Chowdhury, R. *et al.* Highly distinct genetic programs for peripheral nervous system formation in chordates. *BMC biology* **20**, 152 (2022).

7 Brooke, N. M., Garcia-Fernàndez, J. & Holland, P. W. H. The ParaHox gene cluster is an evolutionary sister of the Hox gene cluster. *Nature* **392**, 920-922, doi:10.1038/31933 (1998).

8 Somorjai, I. M. L. *et al.* Wnt evolution and function shuffling in liberal and conservative chordate genomes. *Genome Biol* **19**, 98, doi:10.1186/s13059-018-1468-3 (2018).

9 Wu, H. R. *et al.* Asymmetric localization of germline markers Vasa and Nanos during early development in the amphioxus Branchiostoma floridae. *Dev Biol* **353**, 147-159, doi:10.1016/j.ydbio.2011.02.014 (2011).

10 Shimeld, S. An amphioxus netrin gene is expressed in midline structures during embryonic and larval development. *Dev Genes Evol* **210**, 337-344, doi:10.1007/s004270000073 (2000).

11 Neidert, A. H., Panopoulou, G. & Langeland, J. A. Amphioxus goosecoid and the evolution of the head organizer and prechordal plate. *Evol Dev* **2**, 303-310, doi:10.1046/j.1525-142x.2000.00073.x (2000).

12 Panopoulou, G. D., Clark, M. D., Holland, L. Z., Lehrach, H. & Holland, N. D. AmphiBMP2/4, an amphioxus bone morphogenetic protein closely related to Drosophila decapentaplegic and vertebrate BMP2 and BMP4: insights into evolution of dorsoventral axis specification. *Dev Dyn* **213**, 130-139 (1998).

13 Shimeld, S. M. Characterisation of amphioxus HNF-3 genes: conserved expression in the notochord and floor plate. *Dev Biol* **183**, 74-85, doi:10.1006/dbio.1996.8481 (1997).

14 Somorjai, I., Bertrand, S., Camasses, A., Haguenauer, A. & Escriva, H. Evidence for stasis and not genetic piracy in developmental expression patterns of Branchiostoma lanceolatum and Branchiostoma floridae, two amphioxus species that have evolved independently over the course of 200 Myr. *Dev Genes Evol* **218**, 703-713 (2008).

15 Venkatesh, T. V., Holland, N. D., Holland, L. Z., Su, M. T. & Bodmer, R. Sequence and developmental expression of amphioxus AmphiNk2-1: insights into the evolutionary origin of the vertebrate thyroid gland and forebrain. *Dev Genes Evol* **209**, 254-259, doi:10.1007/s004270050250 (1999).

16 Yu, J. K. *et al.* Axial patterning in cephalochordates and the evolution of the organizer. *Nature* **445**, 613-617 (2007).

17 Pascual-Anaya, J. *et al.* Broken colinearity of the amphioxus Hox cluster. *EvoDevo* **3**, 1-12 (2012).

18 Holland, L. Z., Schubert, M., Kozmik, Z. & Holland, N. D. AmphiPax3/7, an amphioxus paired box gene: insights into chordate myogenesis, neurogenesis, and the possible evolutionary precursor of definitive vertebrate neural crest. *Evolution & development* **1**, 153-165 (1999).

19 Langeland, J. A., Tomsa, J. M., Jackman, W. R., Jr. & Kimmel, C. B. An amphioxus snail gene: expression in paraxial mesoderm and neural plate suggests a conserved role in patterning the chordate embryo. *Dev Genes Evol* **208**, 569-577, doi:10.1007/s004270050216 (1998).

20 Sharman, A., Shimeld, S. M. & Holland, P. An amphioxus Msx gene expressed predominantly in the dorsal neural tube. *Development genes and evolution* **209**, 260-263 (1999).

21 Le Petillon, Y., Oulion, S., Escande, M.-L., Escriva, H. & Bertrand, S. Identification and expression analysis of BMP signaling inhibitors genes of the DAN family in amphioxus. *Gene Expression Patterns* **13**, 377-383 (2013).

22 Jackman, W. R., Langeland, J. A. & Kimmel, C. B. islet reveals segmentation in the Amphioxus hindbrain homolog. *Developmental biology* **220**, 16-26 (2000).

23 Benito-Gutierrez, E., Illas, M., Comella, J. X. & Garcia-Fernandez, J. Outlining the nascent nervous system of Branchiostoma floridae (amphioxus) by the pan-neural marker AmphiElav. *Brain Res Bull* **66**, 518-521 (2005).

24 Holland, L. Z., Schubert, M., Holland, N. D. & Neuman, T. Evolutionary conservation of the presumptive neural plate markers AmphiSox1/2/3 and AmphiNeurogenin in the invertebrate chordate amphioxus. *Dev Biol* **226**, 18-33 (2000).
