# Supplementary material for: An amphioxus neurula stage cell atlas supports a complex scenario for the emergence of vertebrate head mesoderm
Source: Nat Commun. 2024 May 29;15:4550. doi: 10.1038/s41467-024-48774-4 (PMC11136973; doi:10.1038/s41467-024-48774-4)
Supplement: Supplementary file 3 — Description of Additional Supplementary Files [file 41467_2024_48774_MOESM3_ESM.pdf]

Description of Additional Supplementary Files

File Name: Supplementary Data 1
Description: **Shared orthologous markers between chordate developmental cell types and stages (related to Fig. 2).** This table includes genes overexpressed in various cell types of the *B. lanceolatum* neurula transcriptome (reference species 1) and their overexpressed orthologs in other species (species 2 column: *C. intestinalis* or Cint, *X. tropicalis* or Xentro, or *D. rerio* or Drer). For each gene pair, we indicate in which cell type it is overexpressed in each species (and, for the query species 2, which developmental stage); their expression fold change and BH-adjusted enrichment *p*-value (from Wilcoxon rank sum tests) in both species; the gene-name in amphioxus; and whether the gene is a TF or not.

File Name: Supplementary Data 2
Description: **TF binding motifs in *Tbx1/10*, *Gata1/2/3* and *Pitx* candidate enhancers (related to Fig. 5).** List of motifs aligned to each ATAC-seq peak in the vicinity of three regions of interest around the TFs *Tbx1/10*, *Gata1/2/3* and *Pitx*. For each aligned motif, we list the regulatory region where it was found, whether the region was found to drive specific expression in zebrafish embryos ("is expression driver" column; **Fig. 5**), whether the region was tested in zebrafish ("is cloned" column), the motif ID and its annotation based on similarity to known CIS-BP motifs; whether the motif is exclusive to the regulatory region found to drive expression ("is motif exclusive to successful driver?" column), its alignment coordinates along the genome, its alignment score and empirical *p*-value, and the aligned sequence.

File Name: Supplementary Data 3
Description: **Gene family annotation information. a,** Species used for the gene phylogenetic analyses of TFs, including the data sources and their taxonomy. **b,** List of TF families analyzed, including the representative Pfam domains, the *hmmsearch* threshold strategy, and the inflation parameter employed in MCL clustering. **c,** Phylogeny-based classification of amphioxus TFs, with orthogroup names taken from the human orthologs of each gene (using *Possvm*).

File Name: Supplementary Data 4
Description: **Cell type annotation table. a,** Cell type annotations of all metacell clusters in the neurula transcriptome. For each metacell, we indicate its cell type, developmental layer, and whether it has been included in further reclustering analyses. **b-d,** Annotations of metacells for the neural, endodermal and somitic reclustering analyses.

File Name: Supplementary Data 5
Description: Sequences used for probe synthesis and sequence used for mRNA *in vitro* synthesis.

File Name: Supplementary Data 6
Description: Transgenic zebrafish lines data.
