# Supplementary material for: An amphioxus neurula stage cell atlas supports a complex scenario for the emergence of vertebrate head mesoderm
Source: Nat Commun. 2024 May 29;15:4550. doi: 10.1038/s41467-024-48774-4 (PMC11136973; doi:10.1038/s41467-024-48774-4)
Supplement: Supplementary file 5 — Reporting Summary [file 41467_2024_48774_MOESM5_ESM.pdf]

nature portfolio

Corresponding author(s): Hector Escriva

Last updated by author(s): Apr 12, 2024

Reporting Summary

Nature Portfolio wishes to improve the reproducibility of the work that we publish. This form provides structure for consistency and transparency in reporting. For further information on Nature Portfolio policies, see our Editorial Policies and the Editorial Policy Checklist.

Statistics

For all statistical analyses, confirm that the following items are present in the figure legend, table legend, main text, or Methods section.

n/a	Confirmed	
☐	☒	The exact sample size (*n*) for each experimental group/condition, given as a discrete number and unit of measurement
☒	☐	A statement on whether measurements were taken from distinct samples or whether the same sample was measured repeatedly
☐	☒	The statistical test(s) used AND whether they are one- or two-sided<br>*Only common tests should be described solely by name; describe more complex techniques in the Methods section.*
☐	☒	A description of all covariates tested
☐	☒	A description of any assumptions or corrections, such as tests of normality and adjustment for multiple comparisons
☐	☒	A full description of the statistical parameters including central tendency (e.g. means) or other basic estimates (e.g. regression coefficient) AND variation (e.g. standard deviation) or associated estimates of uncertainty (e.g. confidence intervals)
☐	☒	For null hypothesis testing, the test statistic (e.g. *F*, *t*, *r*) with confidence intervals, effect sizes, degrees of freedom and *P* value noted<br>*Give P values as exact values whenever suitable.*
☐	☒	For Bayesian analysis, information on the choice of priors and Markov chain Monte Carlo settings
☐	☒	For hierarchical and complex designs, identification of the appropriate level for tests and full reporting of outcomes
☐	☒	Estimates of effect sizes (e.g. Cohen's *d*, Pearson's *r*), indicating how they were calculated

Our web collection on statistics for biologists contains articles on many of the points above.

Software and code

Policy information about availability of computer code

Data collection: N/A

Data analysis:
STAR v2.7.3
Metacell 0.37
bwa 0.7.17
deeptools 3.5.1 package
biobambam2 2.0.87
MACS2 2.2.7.1
homer 4.11
CIS-BP database
universalmotif 1.12.4
monalisa 1.0 R library
SAMap 1.0.2
scanpy 1.9.3
Broccoli 1.1
HMMER 3.3.2
Pfam 33.0
MCL v14.137
mafft 7.475
IQ-TREE v2.1
ModelFinder

treeshrink v1.3.363
Possvm 1.1
ClustalX

For manuscripts utilizing custom algorithms or software that are central to the research but not yet described in published literature, software must be made available to editors and reviewers. We strongly encourage code deposition in a community repository (e.g. GitHub). See the Nature Portfolio guidelines for submitting code & software for further information.

Data

Policy information about availability of data

All manuscripts must include a data availability statement. This statement should provide the following information, where applicable:

- Accession codes, unique identifiers, or web links for publicly available datasets
- A description of any restrictions on data availability
- For clinical datasets or third party data, please ensure that the statement adheres to our policy

The B. lanceolatum sequencing data generated in this study have been deposited in the GEO database under accession code GSE255742 (https://www.ncbi.nlm.nih.gov/geo/query/acc.cgi?acc=GSE255742), and the corresponding processed gene expression data are available in this same database and the Source Data file. The gene expression data from other chordates, generated in previous studies, are available in the following databases: for Ciona intestinalis GEO database GSE131155; M. musculus, D. rerio and X. tropicalis, TOME database (http://tome.gs.washington.edu/); B. floridae, the publication-specific database (https://lifeomics.shinyapps.io/shinyappmulti/). Accession numbers of sequences used for in situ hybridization probe synthesis are given in Supplementary Data file. Source Data are provided with this paper.

Research involving human participants, their data, or biological material

Policy information about studies with human participants or human data. See also policy information about sex, gender (identity/presentation), and sexual orientation and race, ethnicity and racism.

Reporting on sex and gender	N/A
Reporting on race, ethnicity, or other socially relevant groupings	N/A
Population characteristics	N/A
Recruitment	N/A
Ethics oversight	N/A

Note that full information on the approval of the study protocol must also be provided in the manuscript.

Field-specific reporting

Please select the one below that is the best fit for your research. If you are not sure, read the appropriate sections before making your selection.

☒ Life sciences ☐ Behavioural & social sciences ☐ Ecological, evolutionary & environmental sciences

For a reference copy of the document with all sections, see nature.com/documents/nr-reporting-summary-flat.pdf

Life sciences study design

All studies must disclose on these points even when the disclosure is negative.

Sample size	Sequencing depth and number of libraries were defined to allow support for the paper's main conclusions. In the case of sequencing depth, libraries were sequenced to a median of 6 reads per UMI. The number of cells sorted for transcriptome sequencing was determined based on the expected number of cells in an amphioxus neurula embryo (~3000) and a target coverage (~5x). Concerning in situ hybridization and immunostaining, the sample size is consistent with the habits in the field.
Data exclusions	single-cell transcriptomes deemed to originate from empty MARS-seq wells were removed based on their total UMI counts (see Methods).
Replication	Circa 100 amphioxus embryos were collected at 21hpf, pooled, and used for the single-cell transcriptomics experiments. Sorted cells were sequenced in 40 MARS-seq runs (384 cells in each one). The resulting cell types and metacell clusters were proportionally represented in all sequencing runs. For ISH and immunostaining, n correspond to the number of embryos used, and the micrographs are representative of the pattern observed in all the samples. For the amphioxus embryos injected with Gata1/2/3_Engrailed, injection was undertaken on oocytes from two independant females.
Randomization	The ~100 embryos were randomly selected before sample preparation. For ISH and immunostaining, the embryos were randomly selected from different batches.

Blinding	N/A not applicable to the sc-RNA-seq analysis, nor to the analysis of gene/protein expression by ISH or immunostaining.

Reporting for specific materials, systems and methods

We require information from authors about some types of materials, experimental systems and methods used in many studies. Here, indicate whether each material, system or method listed is relevant to your study. If you are not sure if a list item applies to your research, read the appropriate section before selecting a response.

Materials & experimental systems

n/a	Involved in the study
☐	☒ Antibodies
☒	☐ Eukaryotic cell lines
☒	☐ Palaeontology and archaeology
☐	☒ Animals and other organisms
☒	☐ Clinical data
☒	☐ Dual use research of concern
☒	☐ Plants

Methods

n/a	Involved in the study
☒	☐ ChIP-seq
☒	☐ Flow cytometry
☒	☐ MRI-based neuroimaging

Antibodies

Antibodies used	Anti-laminin antibody (Sigma L9393), Goat anti-Rabbit IgG (H+L) Cross-Adsorbed Secondary Antibody, Alexa Fluor™ 680 (Invitrogen, A-21076)
Validation	The anti-laminin antibody was previously used in PMID: 23201012

Animals and other research organisms

Policy information about studies involving animals; ARRIVE guidelines recommended for reporting animal research, and Sex and Gender in Research

Laboratory animals	Danio rerio (zebrafish) from AB and Tübingen strains were used for embryo obtaining when ages 4 months. Transgenics embryos (F0) were raised until 4 months age for further crossing.
Wild animals	Ripe adults from the Mediterranean amphioxus species (Branchiostoma lanceolatum) were collected at the Racou beach near Argelès-sur-Mer, France, (latitude 42° 32′ 53″ N and longitude 3° 03′ 27″ E) with a specific permission delivered by the Prefect of Region Provence Alpes Côte d'Azur. Branchiostoma lanceolatum is not a protected species. Adults (between 3-7 years old) were sieved from the sand and transferred to the facility and kept in control conditions (light, temperature) during 1 month in small tanks (4L). Gametes were collected by heat stimulation as previously described. Following spawning, adult animals were returned to the sea.
Reporting on sex	Sex was not considered in the study design. For crossings in order to obtain embryos, animal sex was defined on the mrophology of the animals as it is commonly used in all laboratories.
Field-collected samples	No field collected samples were used in this study.
Ethics oversight	All the experiments were performed following the Directive 2010/63/EU of the European parliament and of the council of 22 September 2010 on the protection of animals used for scientific purposes. Ripe adults from the Mediterranean invertebrate amphioxus species were collected with specific permission from the Prefect of Region Provence Alpes Côte d'Azur. Zebrafish embryos were obtained from AB and Tübingen strains, and manipulated following protocols approved by the Ethics Committee of the Andalusia Government and the national and European regulation established.

Note that full information on the approval of the study protocol must also be provided in the manuscript.

Plants

Seed stocks	*Report on the source of all seed stocks or other plant material used. If applicable, state the seed stock centre and catalogue number. If plant specimens were collected from the field, describe the collection location, date and sampling procedures.*
Novel plant genotypes	*Describe the methods by which all novel plant genotypes were produced. This includes those generated by transgenic approaches, gene editing, chemical/radiation-based mutagenesis and hybridization. For transgenic lines, describe the transformation method, the number of independent lines analyzed and the generation upon which experiments were performed. For gene-edited lines, describe the editor used, the endogenous sequence targeted for editing, the targeting guide RNA sequence (if applicable) and how the editor was applied.*
Authentication	*Describe any authentication procedures for each seed stock used or novel genotype generated. Describe any experiments used to assess the effect of a mutation and, where applicable, how potential secondary effects (e.g. second site T-DNA insertions, mosiacism, off-target gene editing) were examined.*
